# Supplementary material for: Tertiary structure and conformational dynamics of the anti-amyloidogenic chaperone DNAJB6b at atomistic resolution
Source: Nat Commun. 2024 Apr 16;15:3285. doi: 10.1038/s41467-024-46587-z (PMC11021509; doi:10.1038/s41467-024-46587-z)
Supplement: Supplementary file 3 — Reporting Summary [file 41467_2024_46587_MOESM3_ESM.pdf]

nature portfolio

Corresponding author(s): Patrick Onck

Last updated by author(s): Jan 31, 2024

Reporting Summary

Nature Portfolio wishes to improve the reproducibility of the work that we publish. This form provides structure for consistency and transparency in reporting. For further information on Nature Portfolio policies, see our Editorial Policies and the Editorial Policy Checklist.

Statistics

For all statistical analyses, confirm that the following items are present in the figure legend, table legend, main text, or Methods section.

n/a	Confirmed	
☐	☒	The exact sample size (*n*) for each experimental group/condition, given as a discrete number and unit of measurement
☐	☒	A statement on whether measurements were taken from distinct samples or whether the same sample was measured repeatedly
☐	☒	The statistical test(s) used AND whether they are one- or two-sided<br>*Only common tests should be described solely by name; describe more complex techniques in the Methods section.*
☒	☐	A description of all covariates tested
☐	☒	A description of any assumptions or corrections, such as tests of normality and adjustment for multiple comparisons
☐	☒	A full description of the statistical parameters including central tendency (e.g. means) or other basic estimates (e.g. regression coefficient) AND variation (e.g. standard deviation) or associated estimates of uncertainty (e.g. confidence intervals)
☐	☒	For null hypothesis testing, the test statistic (e.g. *F*, *t*, *r*) with confidence intervals, effect sizes, degrees of freedom and *P* value noted<br>*Give P values as exact values whenever suitable.*
☒	☐	For Bayesian analysis, information on the choice of priors and Markov chain Monte Carlo settings
☒	☐	For hierarchical and complex designs, identification of the appropriate level for tests and full reporting of outcomes
☒	☐	Estimates of effect sizes (e.g. Cohen's *d*, Pearson's *r*), indicating how they were calculated

Our web collection on statistics for biologists contains articles on many of the points above.

Software and code

Policy information about availability of computer code

Data collection	For performing the molecular dynamics (MD)simulations, an MD engine GROMACS version 2022.3 is used.
Data analysis	Visual Molecular Dynamics (VMD) software is used for the snapshots and analysis. Python, version 3.9 is used for post-processing and plotting.

For manuscripts utilizing custom algorithms or software that are central to the research but not yet described in published literature, software must be made available to editors and reviewers. We strongly encourage code deposition in a community repository (e.g. GitHub). See the Nature Portfolio guidelines for submitting code & software for further information.

Data

Policy information about availability of data

All manuscripts must include a data availability statement. This statement should provide the following information, where applicable:

- Accession codes, unique identifiers, or web links for publicly available datasets
- A description of any restrictions on data availability
- For clinical datasets or third party data, please ensure that the statement adheres to our policy

All data needed to evaluate the conclusions in the paper are present in the paper and/or the Supplementary Information. Data generated from the study is provided in the source data as a file. Initial configuration files for replicating the MD simulations, average structures for closed, open and extended states have been deposited in the Zenodo database under accession code 10592231. Any additional data related to this paper may be requested from the authors.

Research involving human participants, their data, or biological material

Policy information about studies with human participants or human data. See also policy information about sex, gender (identity/presentation), and sexual orientation and race, ethnicity and racism.

Reporting on sex and gender	Not Applicable
Reporting on race, ethnicity, or other socially relevant groupings	Not Applicable
Population characteristics	Not Applicable
Recruitment	Not Applicable
Ethics oversight	Not Applicable

Note that full information on the approval of the study protocol must also be provided in the manuscript.

Field-specific reporting

Please select the one below that is the best fit for your research. If you are not sure, read the appropriate sections before making your selection.

☒ Life sciences ☐ Behavioural & social sciences ☐ Ecological, evolutionary & environmental sciences

For a reference copy of the document with all sections, see nature.com/documents/nr-reporting-summary-flat.pdf

Life sciences study design

All studies must disclose on these points even when the disclosure is negative.

Sample size	Sample size calculation was not performed. Instead, simulations were performed with two different forcefields, CHARMM36m and Amberff99SBdisp is used. For each forcefield three replicas were performed for 2 microseconds each to obtain good sampling of the conformations of the protein for the analysis.
Data exclusions	No data was excluded in performing the analysis
Replication	Three replicas were performed for two different forcefields each to ensure the reproducibility of the data.
Randomization	The initial velocity distribution for every replica is randomized to ensure statistical rigour.
Blinding	Data is generated by GROMACS . The initial configuration of the system, post-processing the data, analysis and drawing conclusions were at least reviewed by 2 people.

Reporting for specific materials, systems and methods

We require information from authors about some types of materials, experimental systems and methods used in many studies. Here, indicate whether each material, system or method listed is relevant to your study. If you are not sure if a list item applies to your research, read the appropriate section before selecting a response.

Materials & experimental systems

n/a	Involved in the study	
☐	☒	Antibodies
☐	☒	Eukaryotic cell lines
☒	☐	Palaeontology and archaeology
☒	☐	Animals and other organisms
☒	☐	Clinical data
☒	☐	Dual use research of concern
☒	☐	Plants

Methods

n/a	Involved in the study	
☒	☐	ChIP-seq
☒	☐	Flow cytometry
☒	☐	MRI-based neuroimaging

Antibodies

Antibodies used	Primary antibodies:<br>Anti-GAPDH: Sigma, Cat No. G8795, Lot 045M4799V, mouse monoclonal, clone GAPDH-71.1, dilution 1:5000 (https://

www.sigmaaldrich.com/NL/en/product/sigma/g8795#product-documentation)

Anti-GFP/YFP: Clontech, Cat No. 632381, Lot A8034133, mouse monoclonal IgG2, clone JL-8, dilution 1:5000 (https://www.takarabio.com/products/antibodies-and-elisa/fluorescent-protein-antibodies/cyan-fluorescent-protein-antibodies)

Anti-V5: Abcam, Cat No. ab9116, Lot 3410457-2, rabbit polyclonal, dilution 1:5000 (https://www.abcam.com/en-nl/products/primary-antibodies/v5-tag-antibody-ab9116)

Secondary antibodies:

HRP-conjugated anti-mouse: GE Healthcare, Cat No. NXA931, Lot 17675041, sheep, dilution 1:5000

HRP-conjugated anti-rabbit: GE Healthcare, Cat No. NA934, Lot 17697725, donkey, dilution 1:5000

Validation

All primary antibodies are validated for detection of the antigen of interest in Western Blot according to manufacturer's websites. Details of the validation statement, antibody profiles and relevant citations can be found on the manufacturer's website.

Eukaryotic cell lines

Policy information about cell lines and Sex and Gender in Research

Cell line source(s)	HEK293T (Human embryonic kidney) cells were obtained from ATCC (Cat. # CRL-3216)
Authentication	HEK293T cells was not authenticated
Mycoplasma contamination	Cell line is regularly checked for mycoplasma infection using PCR twice a year; only mycoplasma-negative cells were used in the study
Commonly misidentified lines (See ICLAC register)	No misidentified cell lines were used
