# Supplementary material for: Tertiary structure and conformational dynamics of the anti-amyloidogenic chaperone DNAJB6b at atomistic resolution
Source: Nat Commun. 2024 Apr 16;15:3285. doi: 10.1038/s41467-024-46587-z (PMC11021509; doi:10.1038/s41467-024-46587-z)

Filter Trap assay (a-c)

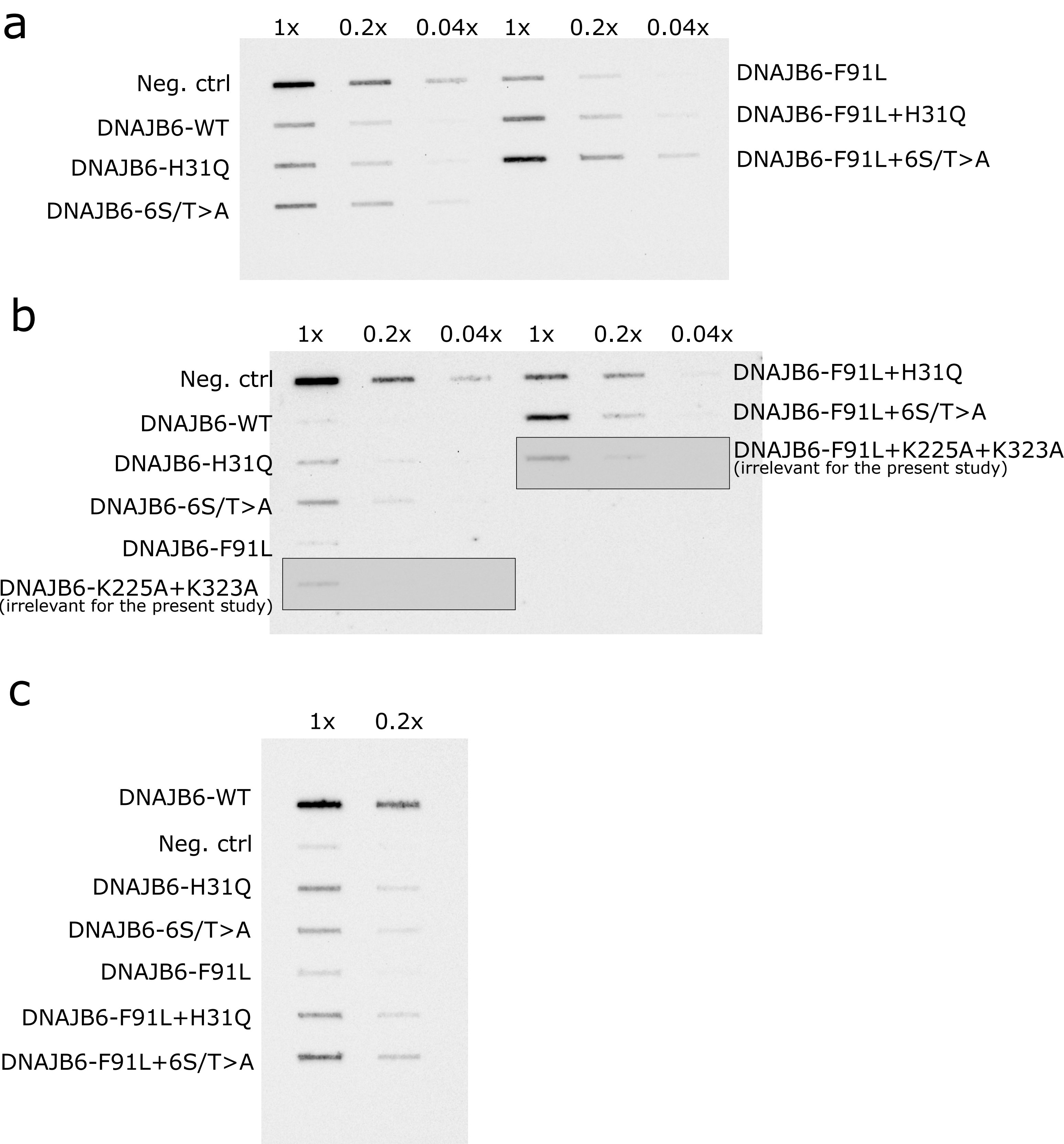

Supplement: Supplementary file 4 — Source Data [file 41467_2024_46587_MOESM4_ESM.zip › Data/Experiments/FTA blots raw.pdf]
